# Supplementary material for: Spatial Structure of NanoFAST in the Apo State and in Complex with its Fluorogen HBR-DOM2
Source: Int J Mol Sci. 2022 Sep 26;23(19):11361. doi: 10.3390/ijms231911361 (PMC9570328; doi:10.3390/ijms231911361)
Supplement: Supplementary file 1 [file ijms-23-11361-s001.zip › ijms-1884029-supplementary.pdf]

Spatial structure of nanoFAST in the apo state and in complex with its fluorogen HBR-DOM2

Vladislav A. Lushpa, Nadezhda S. Baleeva, Sergey A. Goncharuk, Marina V. Goncharuk, Alexander S. Arseniev, Mikhail S. Baranov and Konstantin S. Mineev

Supplementary Materials

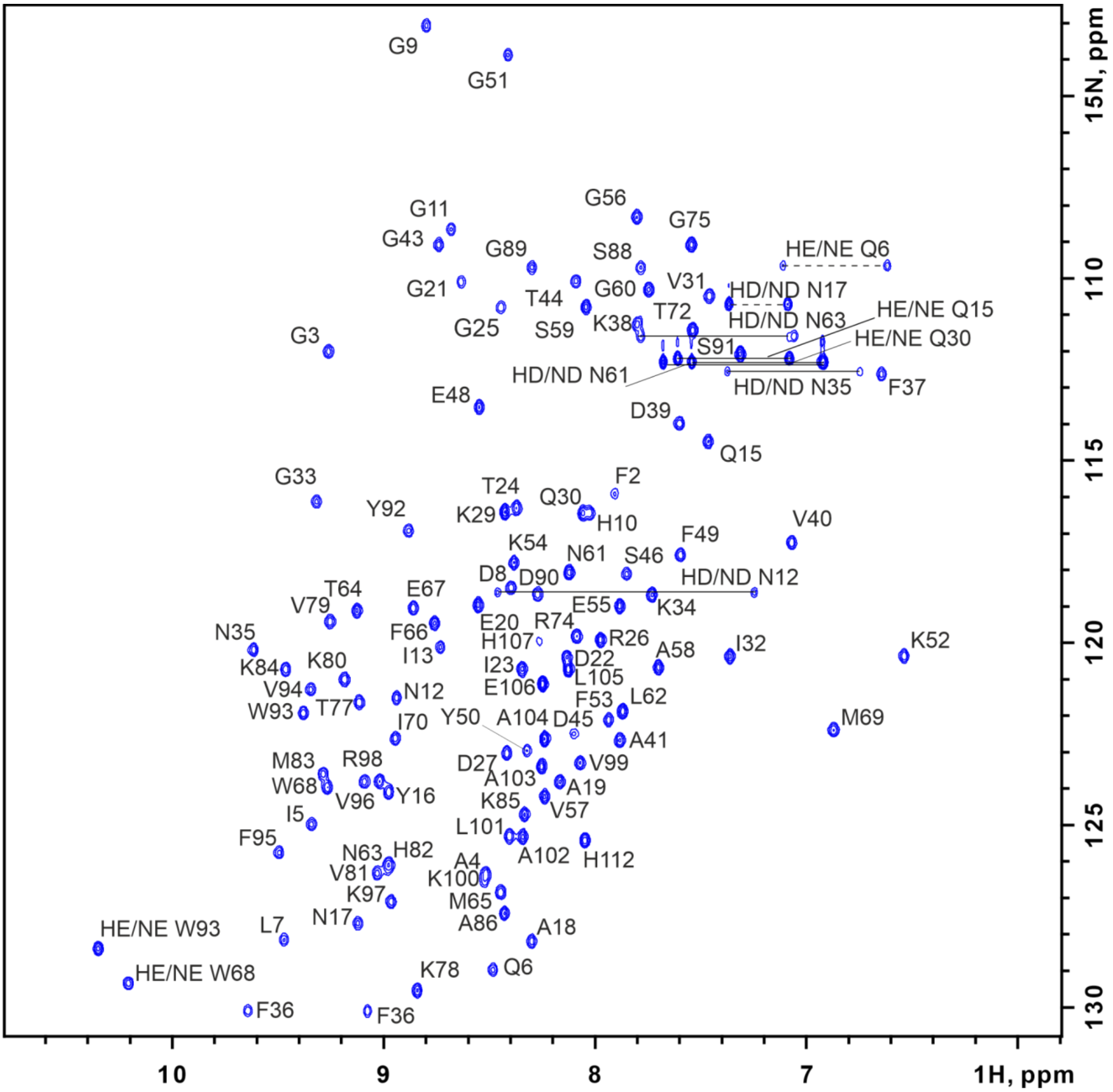

Figure S1. 15N-HSQC spectrum of nanoFAST apo state. Spectrum was obtained at 25 °C, pH 7.0. Assignment of NH groups is indicated.

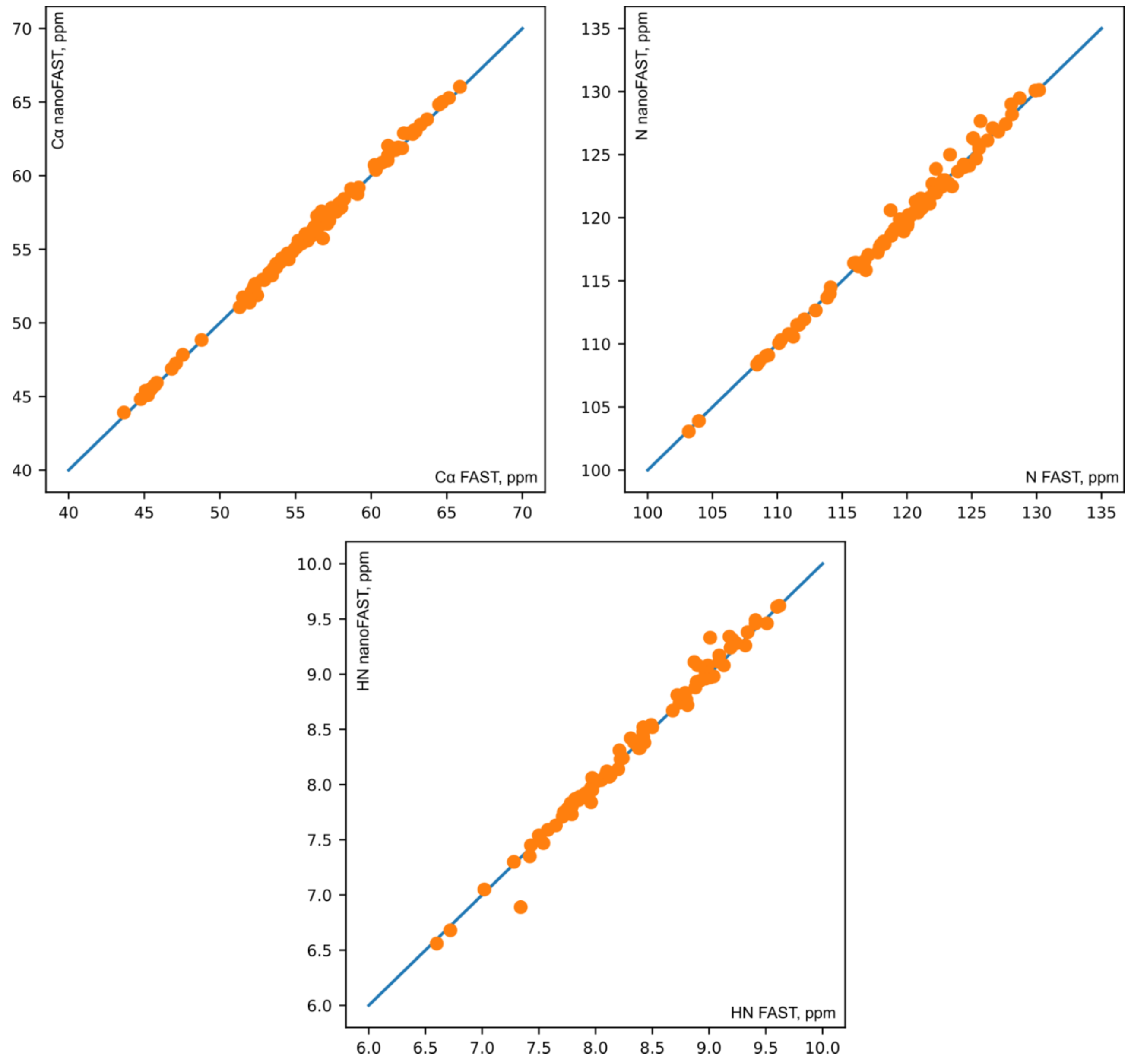

Figure S2. *Chemical shift correlations between FAST and nanoFAST apo states.* Shown are the correlation plots between the ^{13}Cα, ^{15}N and $^{15}H_N$ chemical shifts of nanoFAST and corresponding residues of FAST C-terminal domain. Blue lines show the y=x dependence.

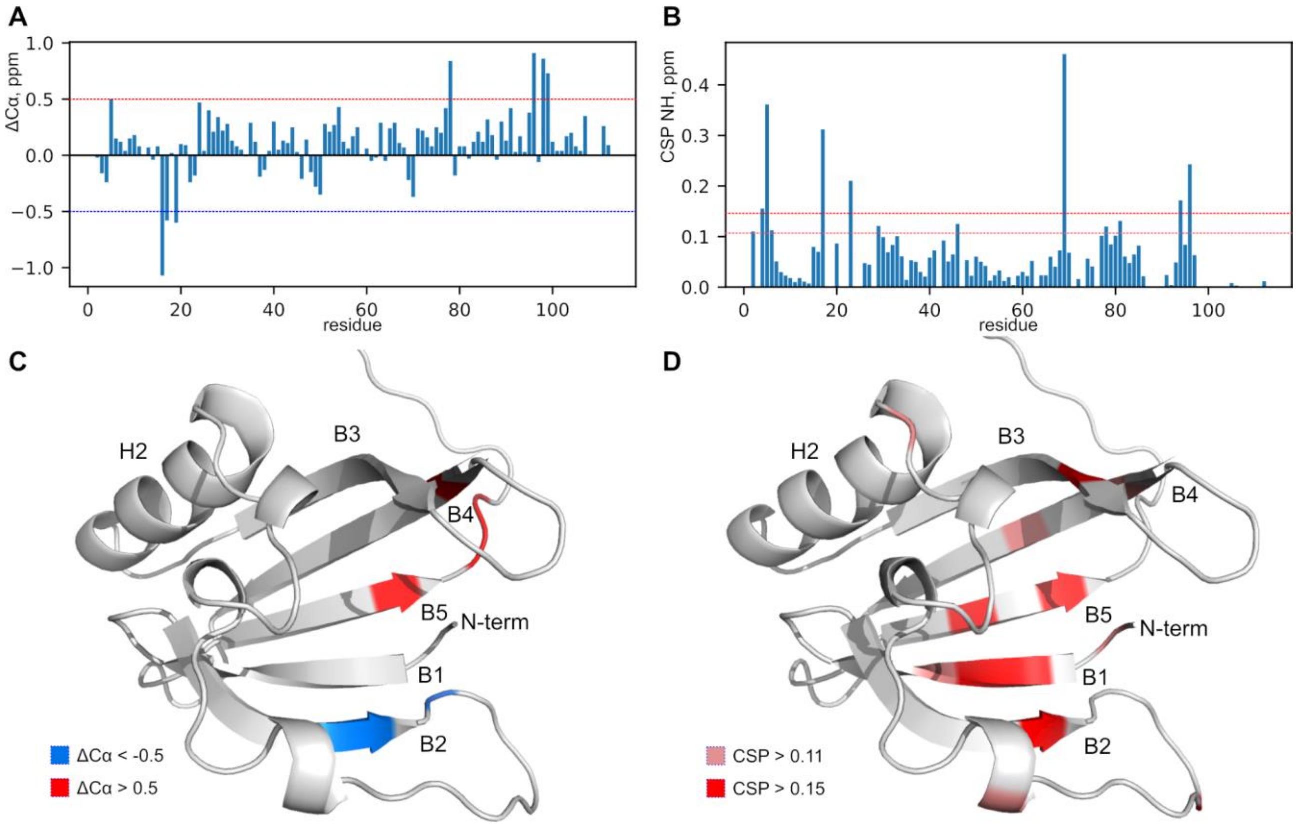

Figure S3. *Chemical shifts differences between FAST and nanoFAST apo states.* **A** - ^{13}Cα chemical shift differences, observed between the residues of nanoFAST and of FAST C-terminal domain in their apo states (nanoFAST-FAST). **B** - Chemical shift perturbations (CSPs) of nanoFAST amide groups, observed due to the absence of the N-terminal domain in the protein. **C,D** - spatial structures of nanoFAST are colored, according to the position of outlying residues on the plots **A** and **B**, respectively. Color codes are provided in the legends.

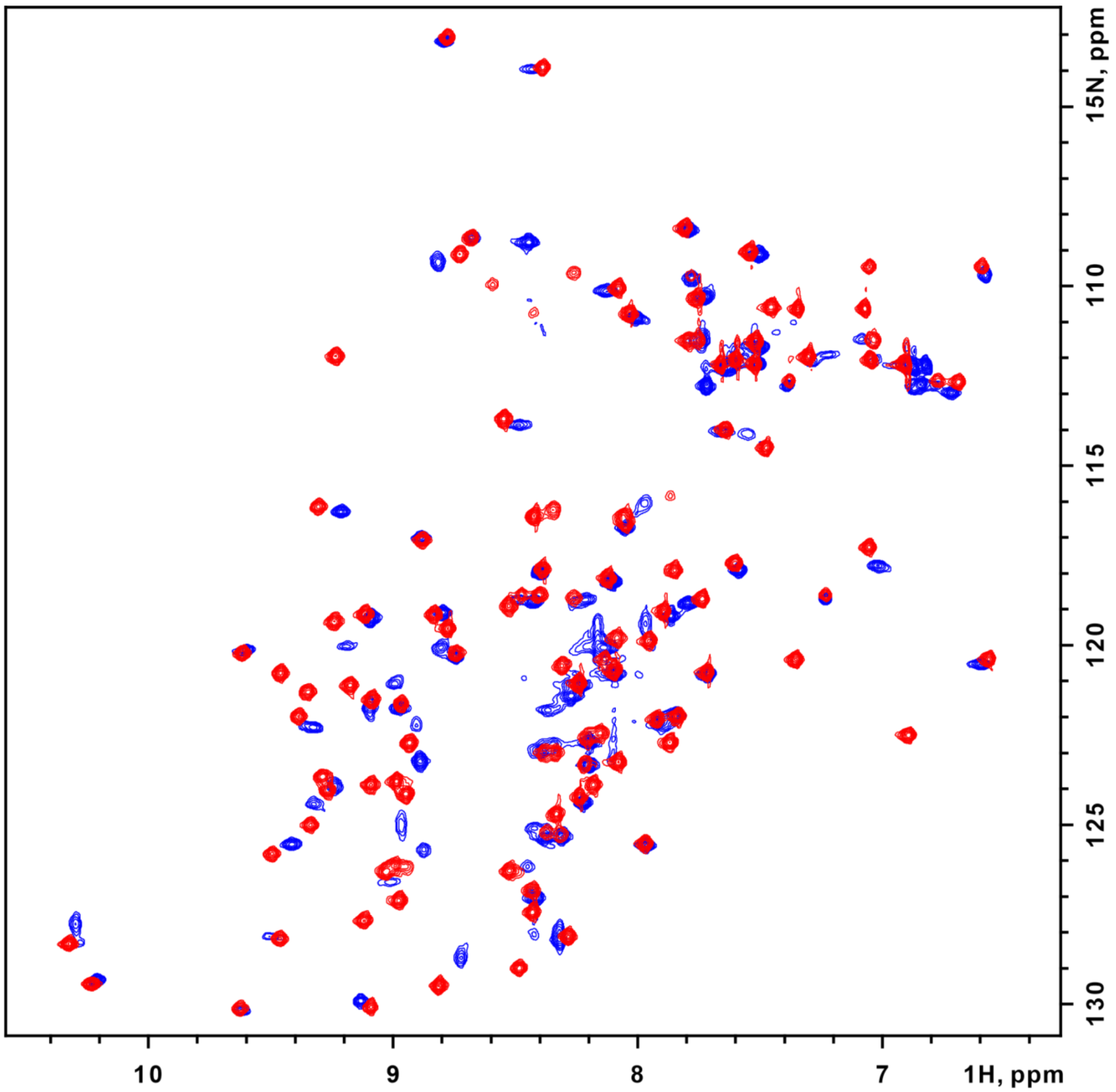

Figure S4. 15N-HSQC spectra of nanoFAST (red) and FAST (blue) apo state. Spectra were obtained at 30 ºC, pH 7.0.

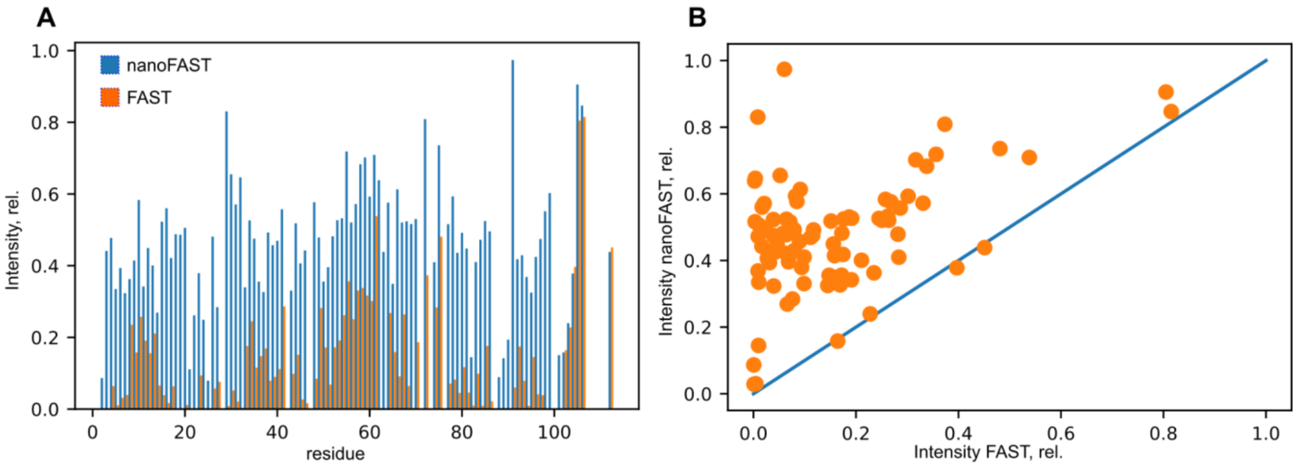

Figure S5. *Intensities of NMR signals in the spectra of nanoFAST and FAST.* **A** - intensities of backbone amide cross-peaks in ^{1}H,^{15}N-HSQC spectra of FAST (shown in orange) and nanoFAST (shown in blue). Spectra were acquired at 30°C, intensities are normalized according to the protein concentrations. **B** - correlation plot of the cross-peak intensities shown in panel **A**. Blue solid line represent the y=x dependence.

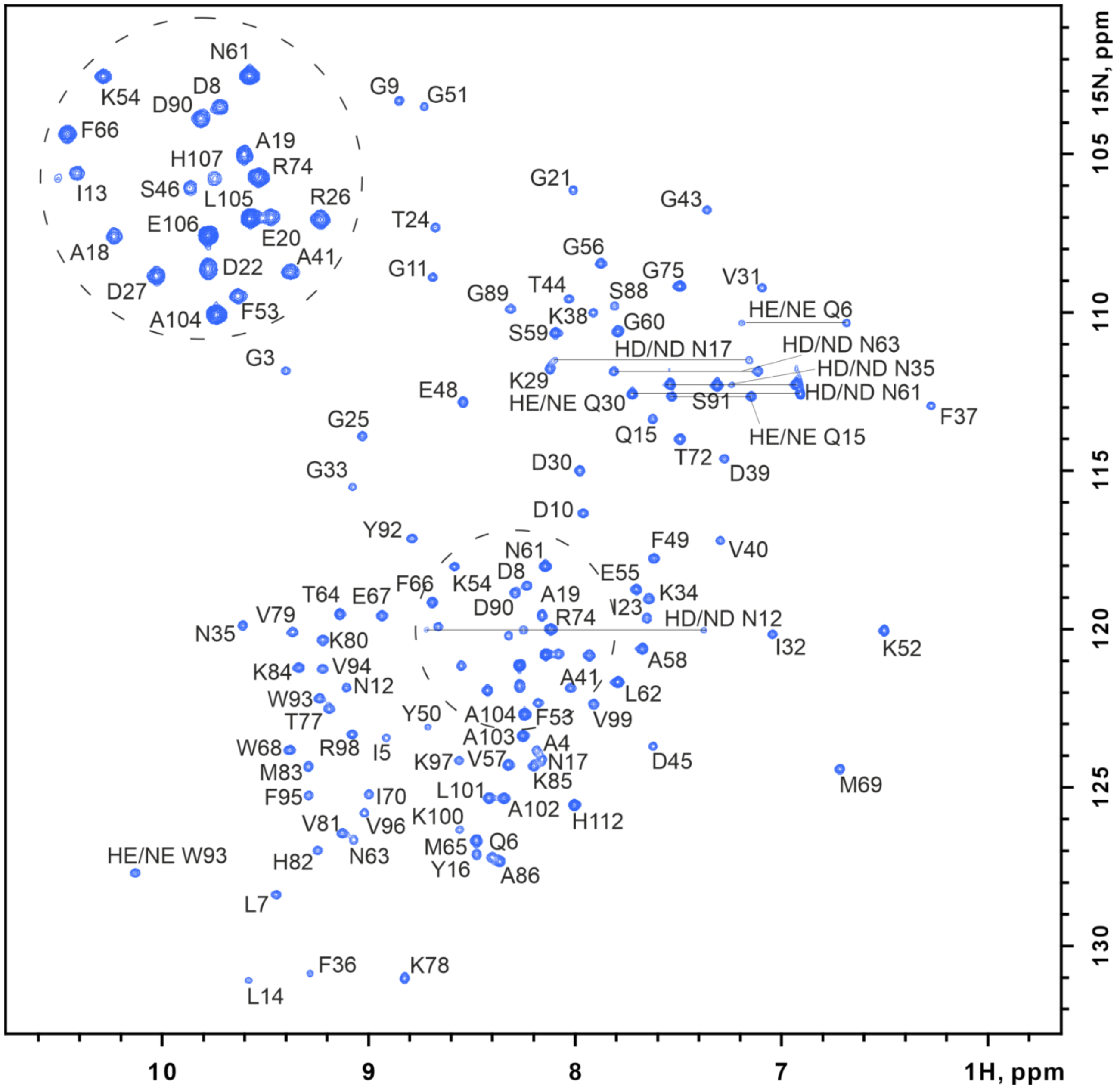

Figure S6. 15N-HSQC spectrum of nanoFAST/HBR-DOM2 complex. Spectrum was obtained at 25 °C, pH 7.0. Assignment of NH groups is indicated.

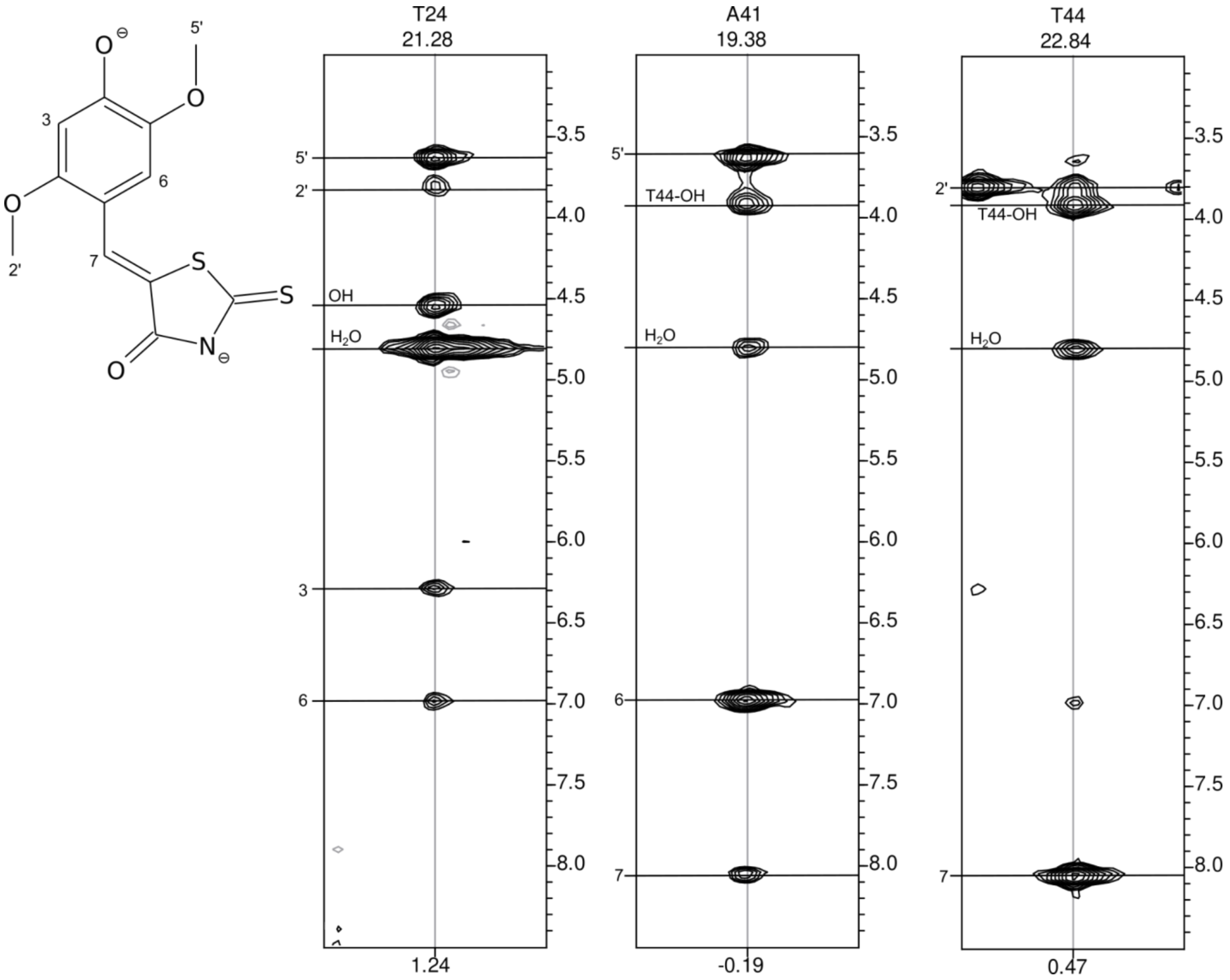

Figure S7. *Ligand-protein interactions in nanoFAST/HBR-DOM2 complex.* Shown are the 2D 1H/1H strips from the 3D 13C/15N-filtered,13C-edited-NOESY-HSQC. Strips correspond to the signals of the T24, A41 and T44 methyl groups, ^{13}C and ^{1}H chemical shifts of the strip anchors are provided at top and at the bottom of the strip, respectively. Assignments of cross-peaks is provided. Structure of HBR-DOM2 with the indicated atom numbering is shown at left.

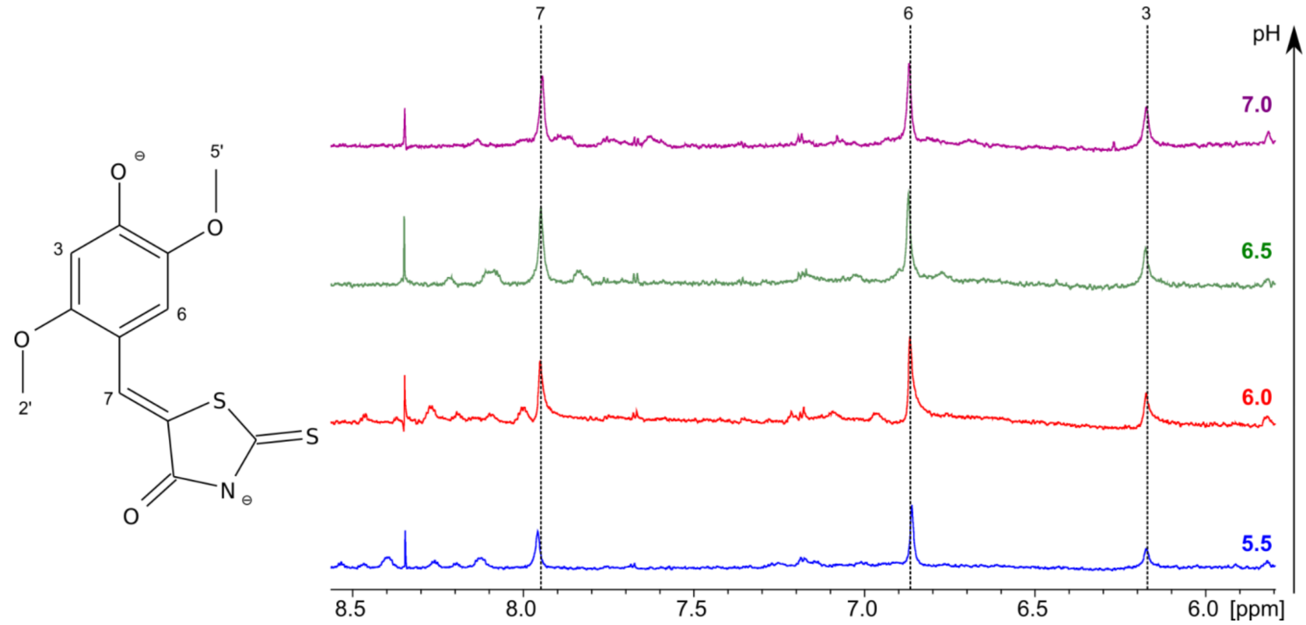

Figure S8. *pH-dependence of HBR-DOM2 NMR spectra.* 1D ^{13}C,^{15}N-filtered NOESY spectra of nanoFAST/HBR-DOM2 complex obtained at various pH in the range 5.5-7.0. Positions of peaks originating from the HBR-DOM2 protons are shown by dashed lines and denoted by atom numbers.

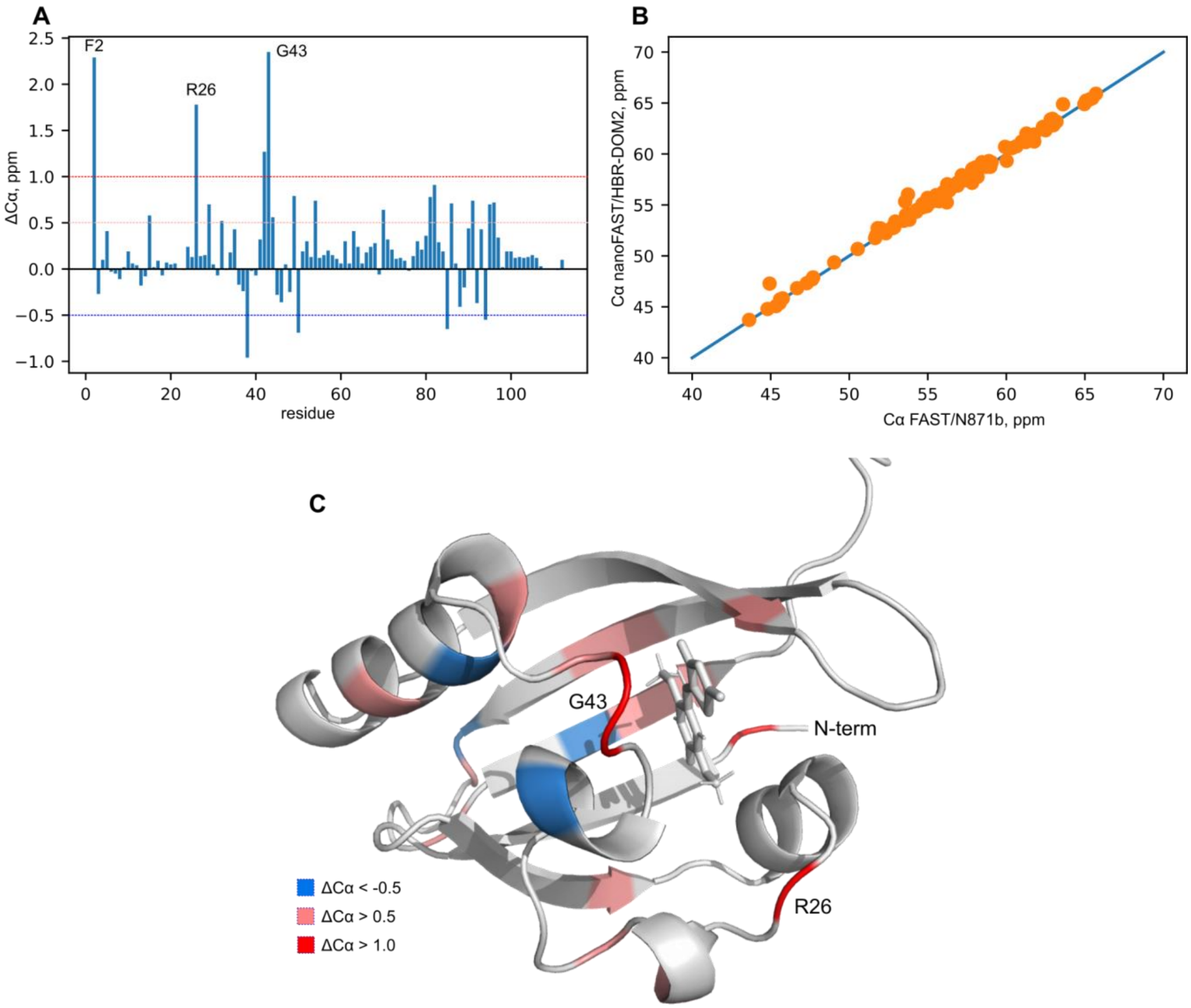

Figure S9. Chemical shift analysis, nanoFAST/HBR-DOM2 vs FAST/N871b. **A** - ^{13}Cα chemical shift differences, observed between the residues of nanoFAST and of FAST C-terminal domain in their complexes with HBR-DOM2 and N871b, respectively (nanoFAST-FAST). Dashed lines denote the thresholds used for the painting in panel **C**. **B** - the correlation plot between the ^{13}Cα chemical shifts of nanoFAST and corresponding residues of FAST C-terminal domain in complexes with fluorogens. Blue lines show the y=x dependence. **C** - spatial structure of nanoFAST/HBR-DOM2 complex is painted according to the chemical shift differences shown in panel **A**.

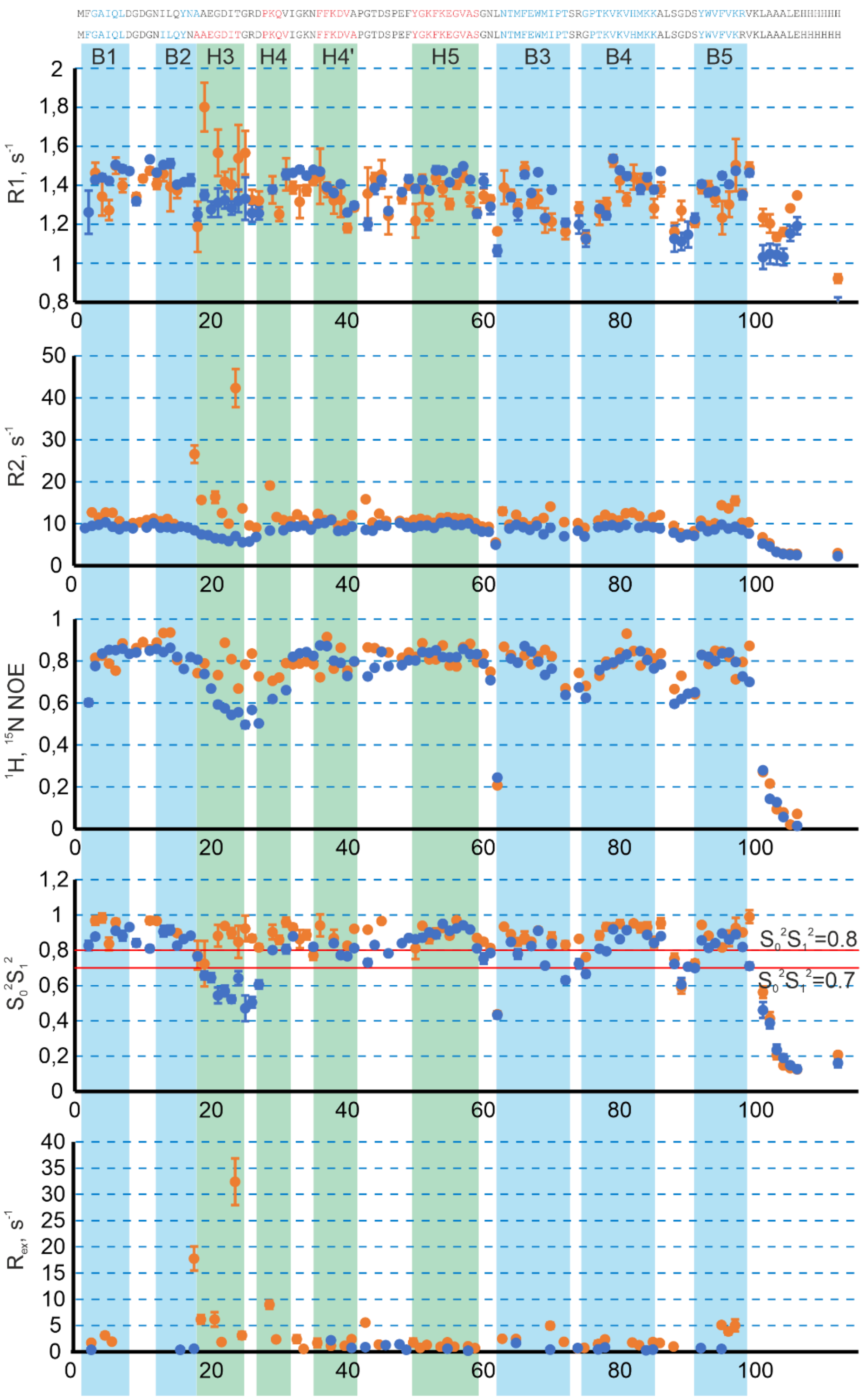

Figure S10. *Dynamics of nanoFAST.* NMR relaxation parameters of ^{15}N nuclei (rates of longitudinal (R1) and transverse (R2) relaxation, heteronuclear equilibrium NOE ($^{1}H,^{15}N$ NOE)) and internal mobility parameters - generalized order parameter $S_0^2S_1^2$ and exchange contribution to the transverse relaxation R_{ex}, measured for nanoFAST in apo state (blue color) and complex of nanoFAST/HBR-DOM2 (orange color).

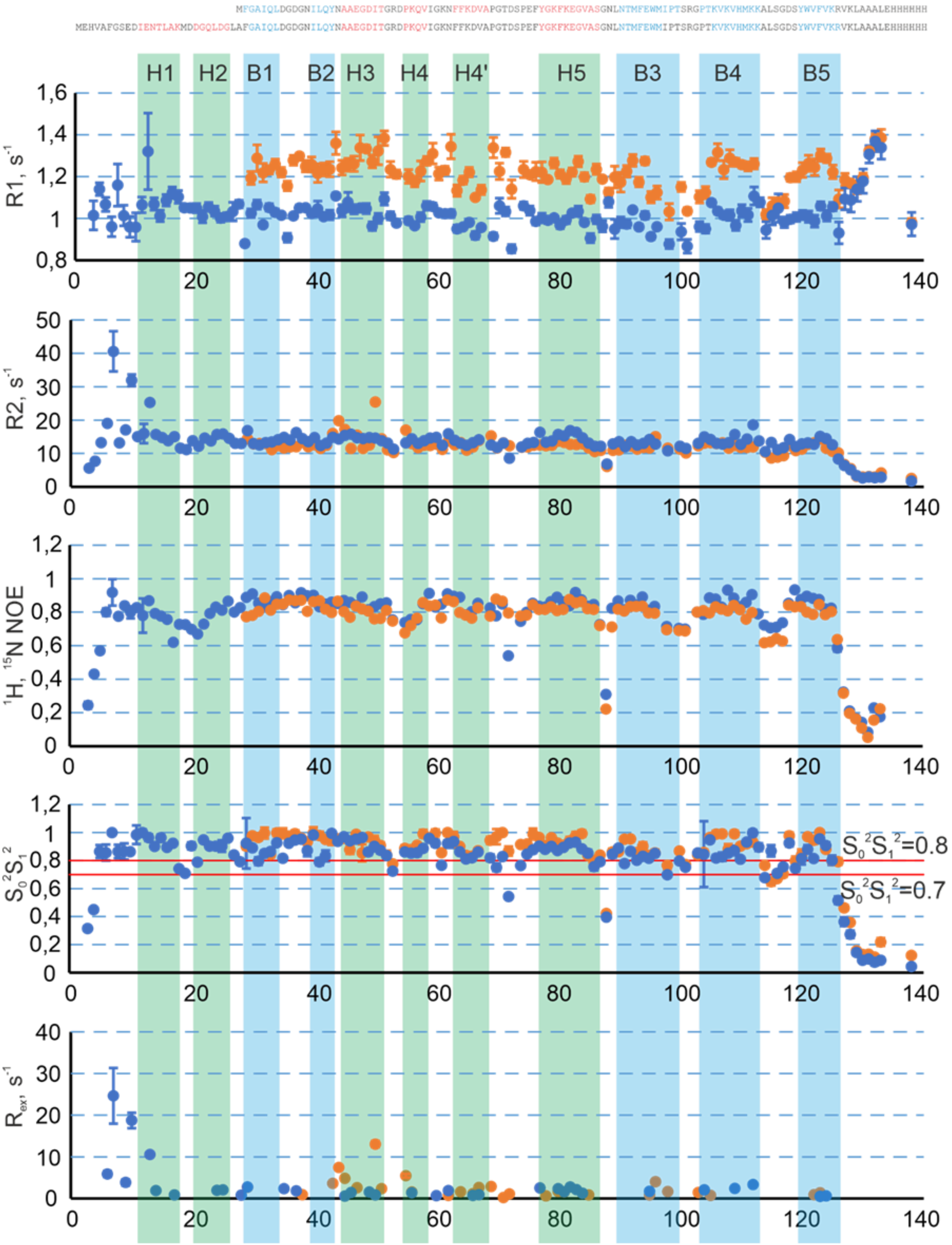

Figure S11. *Comparison of nanoFAST and FAST dynamics.* NMR relaxation parameters of ^{15}N nuclei (rates of longitudinal (R1) and transverse (R2) relaxation, heteronuclear equilibrium NOE (^{1}H,^{15}N NOE)) and internal mobility parameters - generalized order parameter $S_0^2S_1^2$ and exchange contribution to the transverse relaxation R_{ex}, measured for nanoFAST/HBR-DOM2 (orange color) and FAST/N871b (blue color) complexes. Numeration in nanoFAST is shifted by 27 residues.

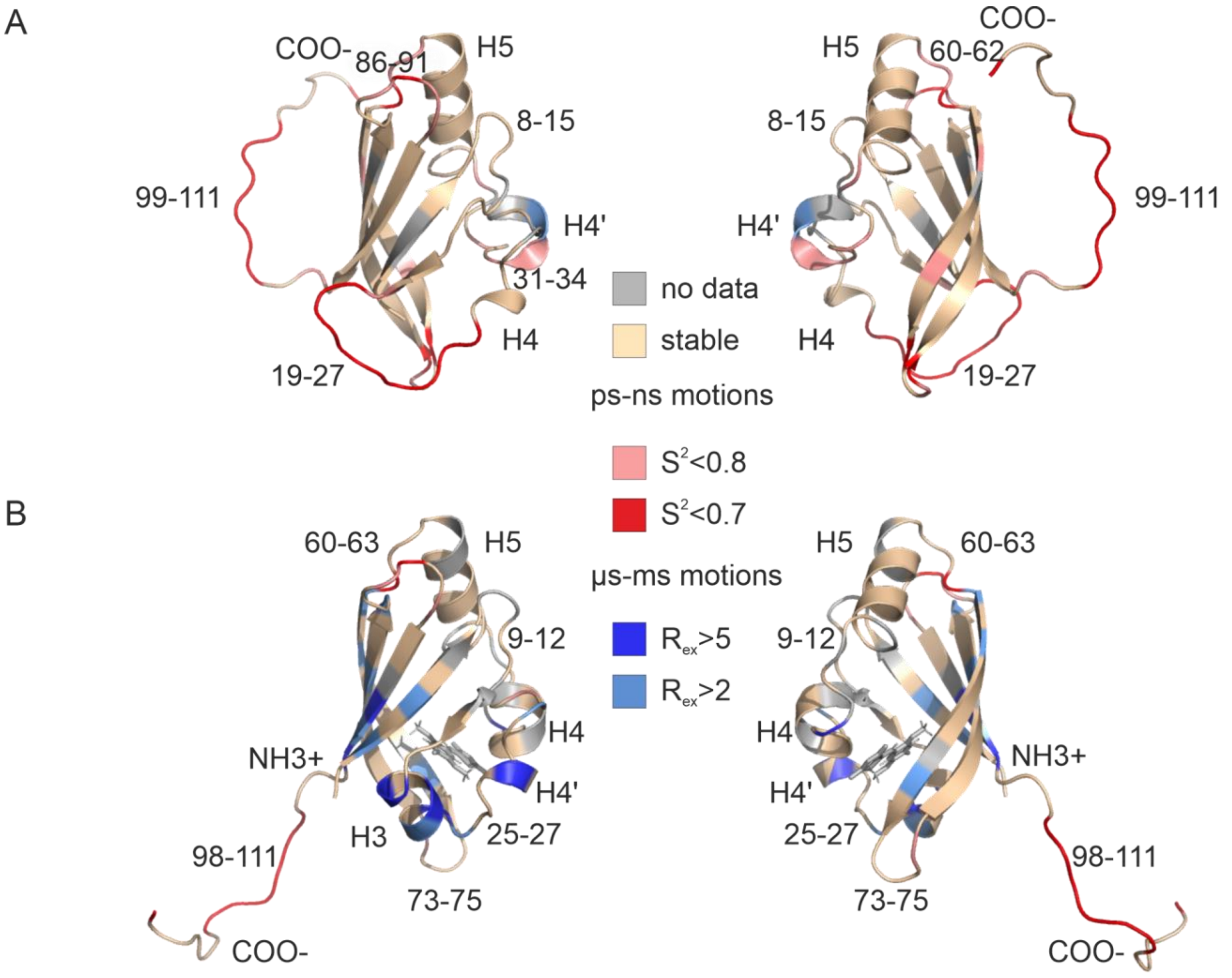

Figure S12. Motions of the protein backbone. A - motions of the backbone of nanoFAST in apo state. **B** - motions of the backbone of nanoFAST in complex with HBR-DOM2. Color codes are provided in the legends.

HBR-DOM2 **N1036** **N1048**

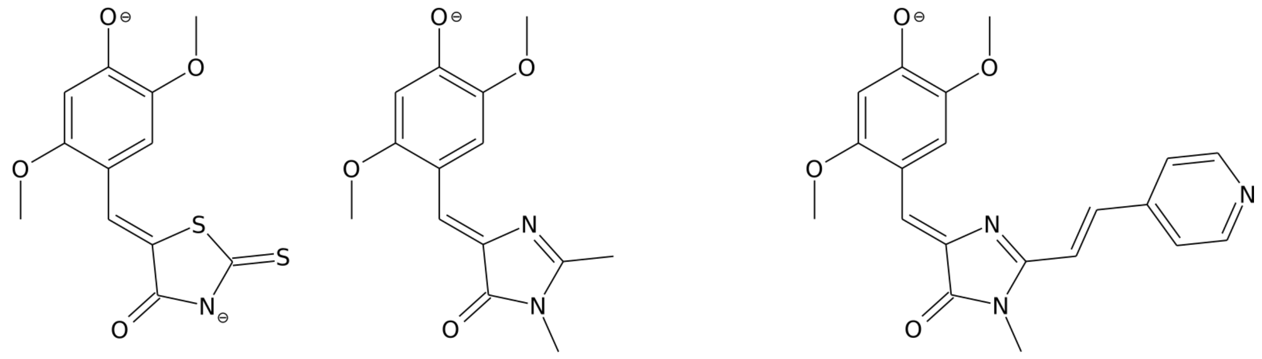

FAST Kd: 21 nM 710 nM 150 nM

Figure S13 Structures and [FAST-fluorogen] dissociation constants of HBR and HBI ligands with the same substitutients of 4-hydroxybenzyldiene group. Kds are provided according to the work [11] (HBR-DOM2) and according to the current work (N 1036 and N 1048).

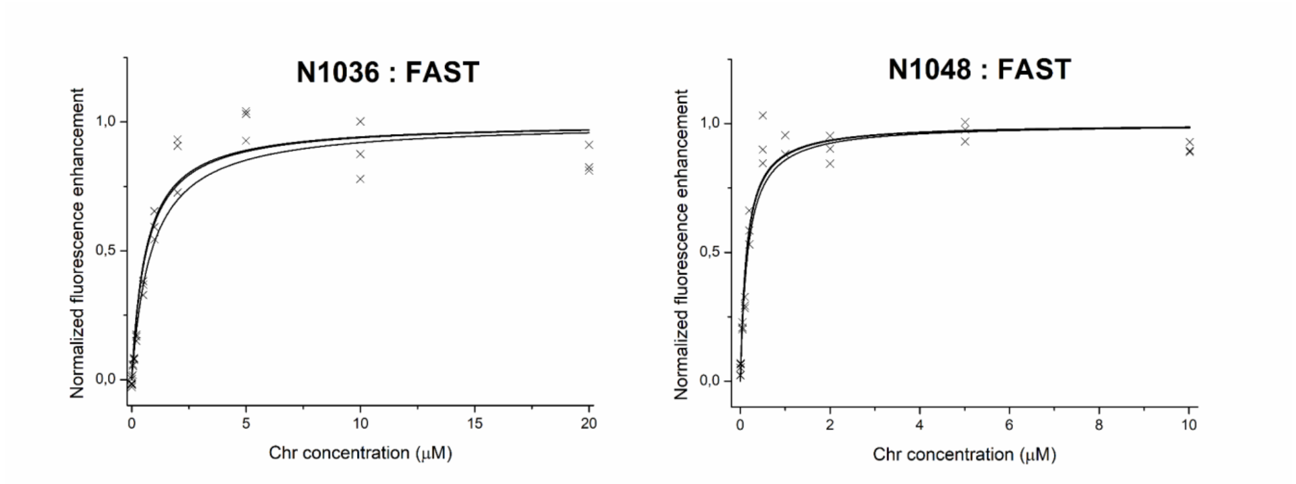

Figure S14. Titration curves observed for **N 1036** and **N 1048** complexes with FAST.

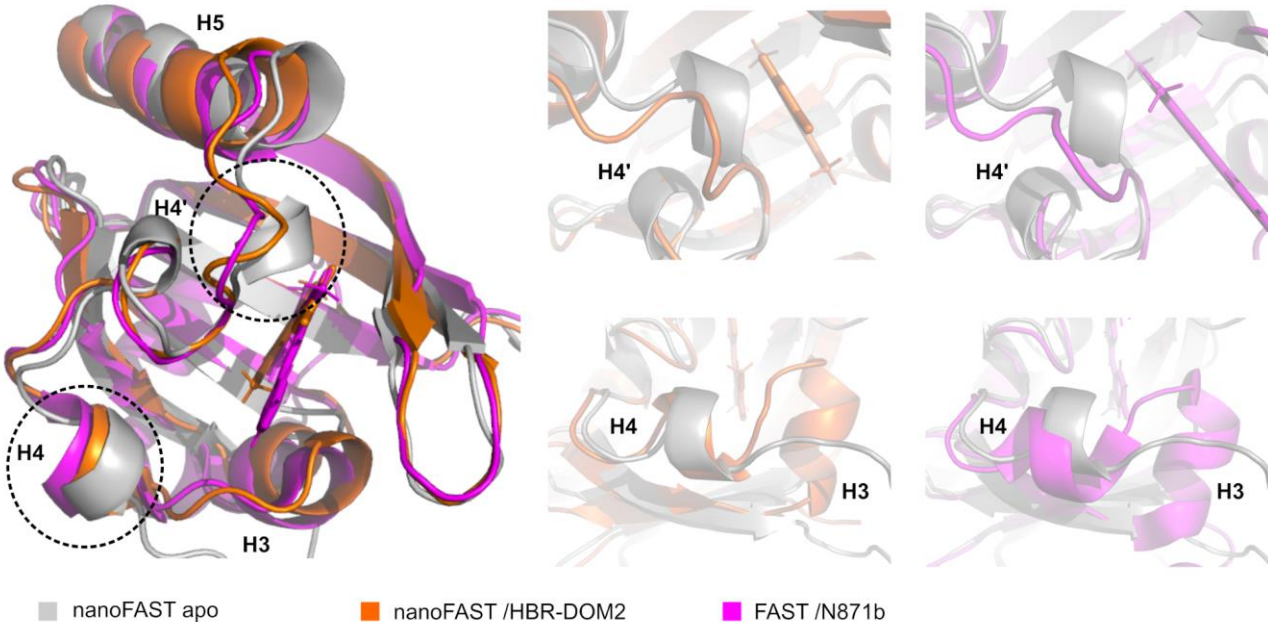

Figure S15. Ligand-induced changes in the nanoFAST structure. Shown are the spatial structures of nanoFAST apo state (gray), nanoFAST/HBR-DOM2 (orange), FAST/N871b (magenta). Structures are superimposed using the backbone atoms of the β-sheet residues. Zoomed views on the regions, shown by black circles are provided in right panels.

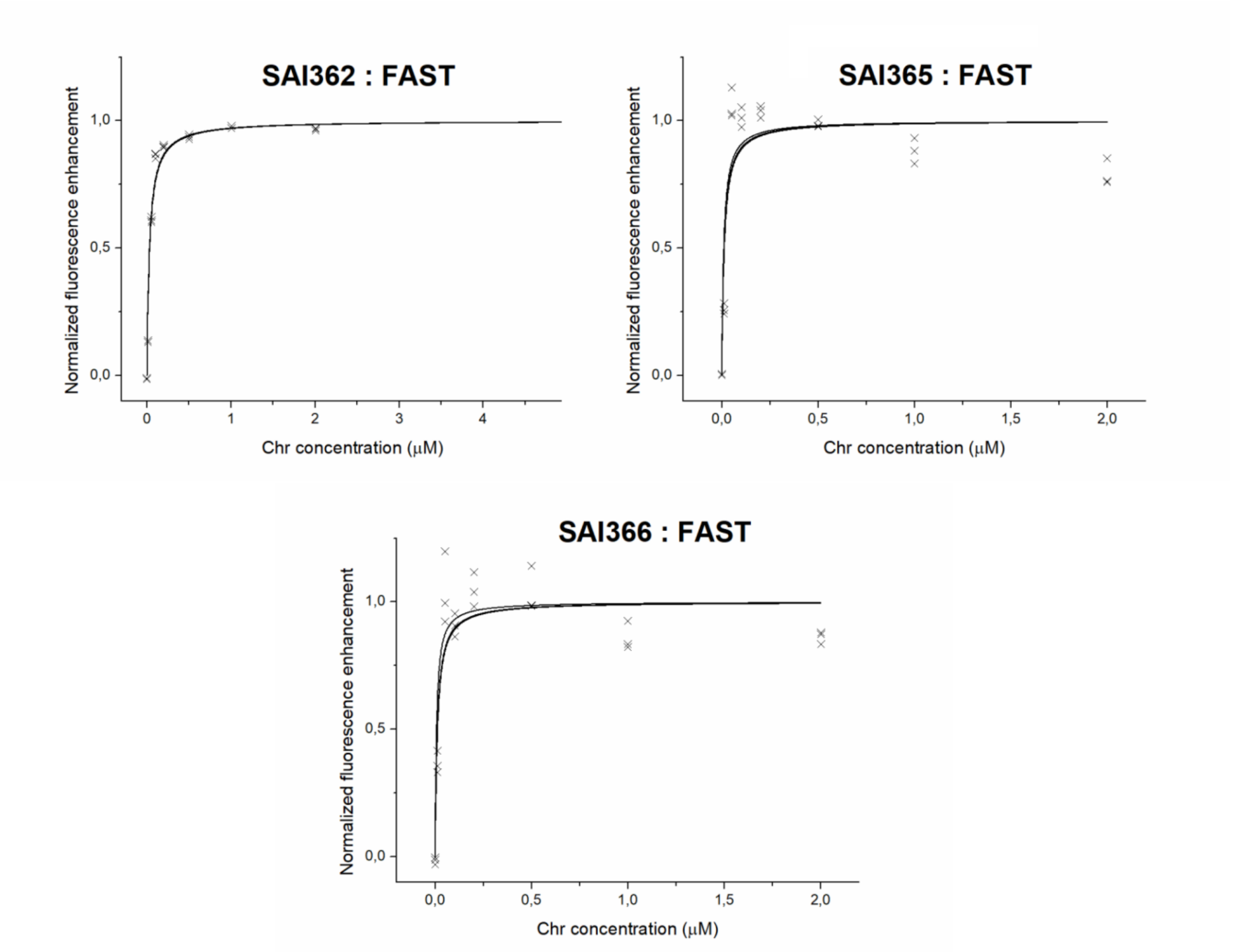

Figure S16. Titration curves observed for **SAI 362**, **SAI 365** and **SAI 366** complexes with FAST. Lines indicate the result of approximation.

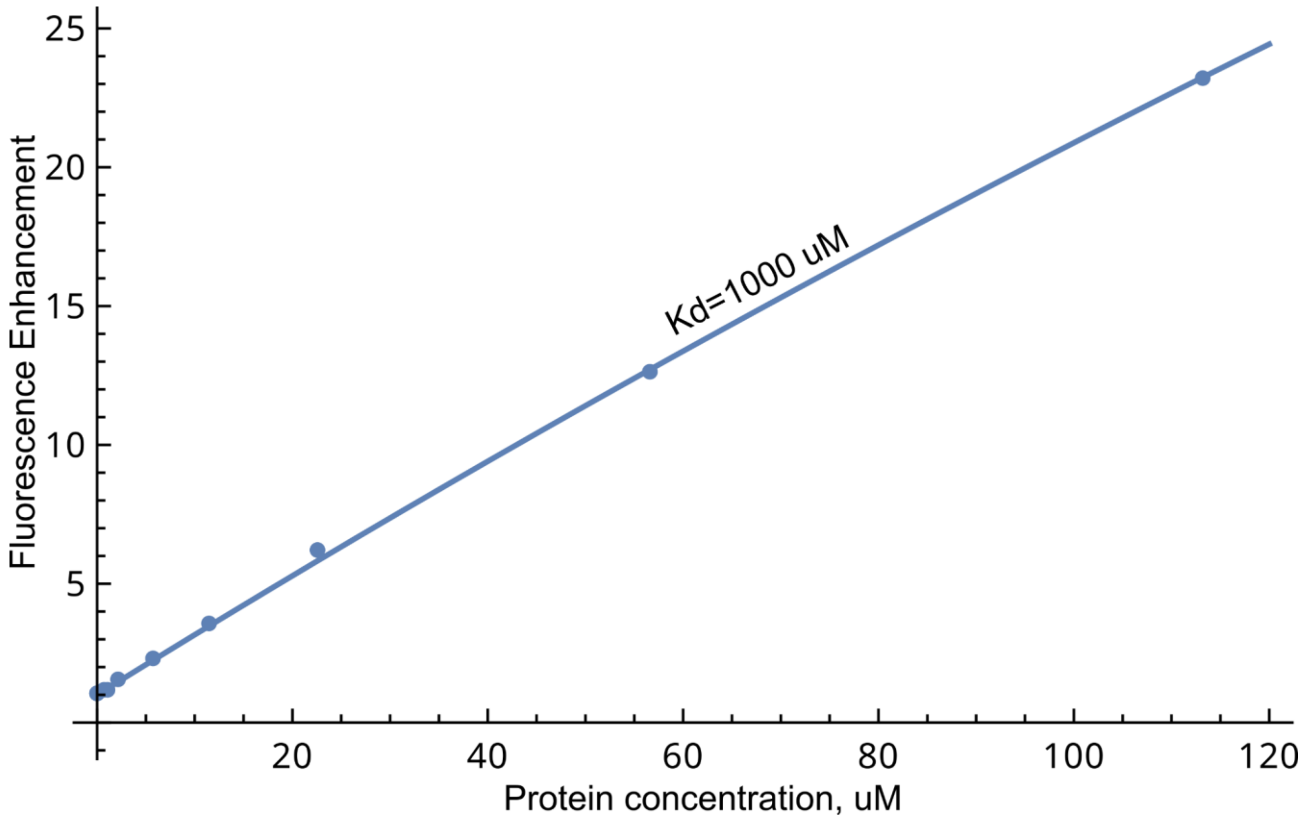

Figure S17. Titration curve, observed for the N871b complex with nanoFAST. Line denotes the result of the data approximation assuming the Kd equal to 1 mM.

Table S1. Dissociation constants values of complexes [FAST-chromophore]

Chromophore	Structure	K_{D}, µM
**SAI 362**	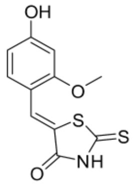	0.031±0.001
**SAI 365**	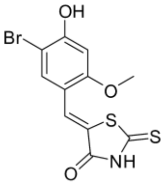	0.012±0.001
**SAI 366**	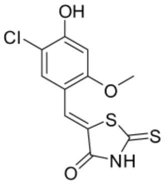	0.010±0.003
**N 1036**	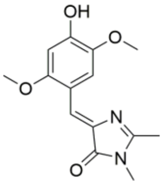	0.71±0.14
**N1048**	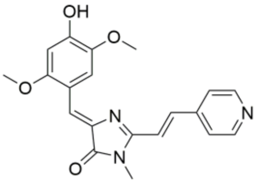	0.15±0.01
